# The Future of Disease Control Priorities: Comment on "Disease Control Priorities Third Edition Is Published: A Theory of Change Is Needed for Translating Evidence to Health Policy"

**DOI:** 10.15171/ijhpm.2018.119

**Published:** 2018-12-10

**Authors:** Prabhat Jha

**Affiliations:** Centre for Global Health Research, St. Michael’s Hospital and Dalla Lana School of Public Health, University of Toronto, Toronto, ON, Canada.

**Keywords:** Priority Setting in Health, Economic Evaluation, Global Burden of Disease, Direct Mortality Measurement

## Abstract

The Disease Control Priorities (DCP) project has substantially influenced national and global health priorities since 1993. DCP’s basic framework involves identification of disease burdens based on premature deaths and disability and application of the most cost-effective interventions to the largest burdens, taking into account local feasibility. The future impact of DCP will need to take into account growing national wealth and needs for endogenous capacity to design and implement evidence-based interventions, the rapid emergence of non-communicable disease (NCD), and the universal health coverage (UHC) agenda. This in turn requires three improvements to the DCP framework: greater local capacity, supported by a global effort to cost health interventions, stronger national and international technical capacity and networks, and the use of direct, versus modelled, mortality data to assign priorities and to assess progress. Properly done, DCP could be as important over the next 25 years as it has been in the past 25 years.

## The Future of Disease Control Priorities

For much of the last 25 years, the framework set out in the Disease Control Priorities (DCP) project has influenced global health directions. The basic framework is to identify disease burdens based on premature deaths and disability, and then apply the most cost-effective interventions to the largest burdens, taking into account local feasibility. These are assembled into priority “packages” that can be introduced at the country level, as illustrated from DCP application to India (Figure).^1^ DCP1^2^ informed the World Bank’s influential World Development Report (WDR) *Investing in Health.*^3^ DCP2 followed in 2006 and deepened the analyses of health systems.^4^ DCP3, published during 2014-2017, expanded the methodology to focus on poverty reduction and to ensure recommended packages aligned with the World Health Organization’s (WHO’s) plans for universal health coverage (UHC).^5^ DCP3 is thus particularly relevant to various disease- and poverty-specific sub-goals within the United Nations Sustainable Development Goals.^6^

**Figure:**
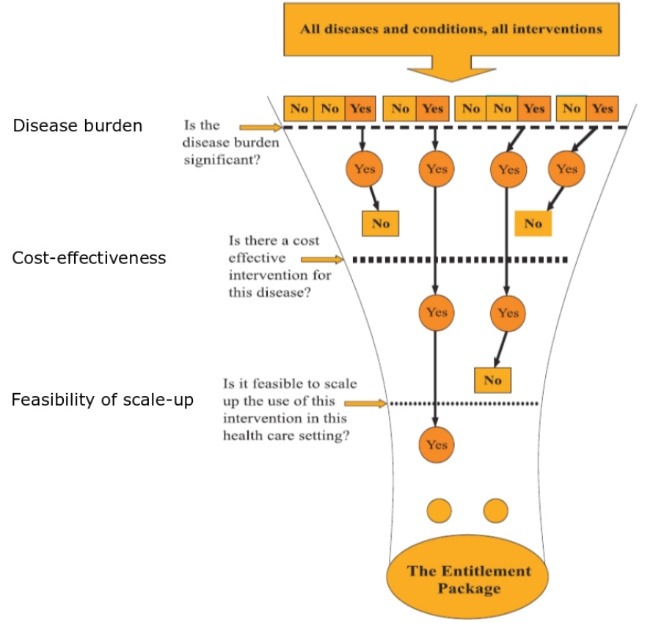
Design of Priority Intervention (“Entitlement”) Packages in India to Tackle Major Causes of Premature Mortality.^1^

DCP’s influence, as reviewed by Ole Norheim,^7^ has been wide and deep on country applications and global health priority setting. Norheim lays out a Theory of Change that would increase the use of DCP methods at the country level. To supplement his useful ideas, I comment on the impact of DCP and the complementary role of reliable and direct measures of disease burden. I provide three directions for better use of evidence at the global and at the country level.

## Impact of Disease Control Priorities

DCP1 informed the design of a $1 billion portfolio of World Bank-assisted state-level health systems and categorical disease control programs in India.^8^ More recently, the DCP2 methodology has been applied nationally^1^ and for the state of Karnataka,^9^ and is influencing the debate on introduction of UHC. Similarly, DCP1 influenced the design of Mexico’s healthcare reforms.^10^ Norheim and Dean Jamison, the lead author of the WDR and chief editor of DCP, both point out the more limited uptake of DCP in other low- and middle-income countries (LMICs). The main constraint is inadequate technical capacity in many LMICs to generate and use the DCP methodology.^5,7^ Less equivocally, DCP1 motivated Bill and Melinda Gates to invest in global health through the Bill & Melinda Gates Foundation.^11^ DCP1, paired later with the Commission on Macroeconomics and Health, played a role in catalysing the Global Fund to Fight AIDS, Tuberculosis and Malaria.^12^

Quantifying the impact of DCP1 and DCP2 is difficult, given problems attributing impact to disease control programs recommended by DCP. An interesting, albeit limited, comparison is of the equal value for money of a $50 million investment on HIV control in India by the Government of India and the World Bank (beginning in 1999) when compared to the Gates Foundation’s Avahan, which spent about 5 times more (and began later). India’s Second National HIV/AIDS Program drew broadly on DCP1 work led by Peter Piot and others^2^ that recommended prevention and treatment of sexually-transmitted infection in female sex workers as being particularly cost-effective. A quantitative analysis showed that each additional sexually-transmitted infection treated in India appeared to reduce the risk of HIV or syphilis infection in pregnant women from 2003-2008 (as a surrogate of HIV infection in the general population). Importantly, there was no difference between the cheaper Government of India-funded and more expensive Avahan-funded programs in the reductions in HIV or syphilis.^13^

## Reliable Mortality Burdens

An early innovation in the DCP methodology was to estimate disease burdens alongside economic analyses. Jamison conceived the idea of the Global Burden of Disease (GBD) in the early 1990s, drawing upon Alan Lopez’s assembly of consistent estimates of death by cause worldwide, the Richard Zeckhauser and Don Shepard metric that combined fatal and non-fatal health events (which led to the disability-adjusted life year, or DALY), and Howard Barnum’s illustration of national burden of disease in Ghana that combined non-fatal outcomes with consistent cause of death estimates. Jamison contracted Harvard University to make calculations for WDR, which resulted in the first (1990) GBD estimate.^12^ Lopez and Chris Murray have subsequently expanded the GBD markedly.^14^

At the global level, the 1990 (and 2000) GBD was an important advance, mostly in ensuring consistent estimates of the causes of death for the world and for major regions.^4,12^ However, subsequent estimates for the national level have been problematic.^15^ This is because the evidence on causes of death, and to a lesser extent on levels of mortality, in the GBD are based far more on econometric models than on actual data (and indeed often on models of models). Not surprisingly, this leads to odd results. For example, the modelled data stated that child mortality was rising by 3% a year in South Africa, whereas direct data from local census and statistical agencies found that it was falling at 3% a year.^16^ The estimation of the burden of causal risk factors (which is particularly needed for control of non-communicable diseases [NCDs]) is also challenging. India’s ranking of causal risk factors was markedly different between 2010 and 2015 (Table).^14^

**Table:** Differences Between GBD Rankings of India’s DALYs Lost (a Combined Measure of Premature Mortality and Serious Disability) for 2010 and 2015

	**Indian DALYs: 2010 Ranking**		**Indian DALYs: 2015 Ranking**
1	Diet	1	Blood pressure
2	Household air pollution	2	Fasting plasma glucose
3	Smoking	3	Ambient particulates
4	Blood pressure	4	Household air pollution
5	Childhood underweight	5	Unsafe water
6	Occupational risks	6	Childhood undernutrition
7	Ambient particulates	7	Smoking
8	Fasting plasma glucose	8	Total cholesterol
9	Iron deficiency	9	Iron deficiency
10	Alcohol	10	Diet lacking whole grains

Abbreviations: GBD, Global Burden of Disease; DALYs, disability-adjusted life years. These temporal changes in rankings are mostly due to changes in model assumptions, not in the risk factors themselves. Source: author calculations based on GBD.^14^

## The Way Forward

DCP3’s impact will materialize over the next few years. Three major factors are likely to influence uptake of DCP evidence: the growing wealth of many LMICs, the emergence of NCDs as the main challenge facing many countries, and the priority laid out by WHO to advance UHC and the related World Bank agenda to improve human capital (which includes both child and adult mortality, along with nutrition and educational achievement).^17^

Rising wealth means that many LMICs can now implement interventions that they would not have considered earlier. Naturally, expectations are clear that Ministries of Health, and not donors or Western universities, should drive the evidence agenda. The emergence of NCDs implies that far more reliable country-level quantification of risk factors is required than that produced by the currently unreliable models, paired with strategies to scale up NCD prevention and treatment. The obvious priorities are large increases in tobacco taxation and other tobacco control efforts, combined with access to low-cost treatment of curable or treatable common vascular conditions and cancers.^5,12,18^ At the national level, DCP can help design intervention packages for UHC. For example, the Ayushman Bharat program in India, which aims to expand health insurance to 500 million Indians, has broadly adopted cost-effectiveness and use of treatment packages.^19^

Three changes in the DCP approach are required to meet the changing landscape of global health, and to ensure relevance to countries. First, a substantial investment is needed to build on the DCP3 work, in particular in costing various health interventions (individual or combined) in different settings with markedly dissimilar cost structures. In particular, these will need to pay attention to the community and population-based strategies to tackle the risk factors which are emerging from epidemiological studies in LMICs (such as the unique role of low body weight in explaining the high risks of vascular disease in India).^20,21^ These data should be shared openly and publically for unrestricted use on a globally accessible website. A large costing platform, building upon the WHO’s earlier “CHOICE” analyses would substantially reduce one of the lingering uncertainties in the design of intervention packages.^5^ Second, as Norheim argues,^7^ much stronger technical capacity is needed, including creation of national public or voluntary institutions to do rational planning and analyses free from the vagaries of daily fire-fighting that is all too common for most ministries of health. Such efforts are commensurate with building national country capacity for governance and monitoring. Global networks of DCP practitioners, akin to successful clinical epidemiological networks, are needed to increase use and uptake in many LMICs. A DCP network would be a good investment.

Third, the design of DCP intervention packages and the monitoring of their impact require far more reliance on direct mortality data than on models. Direct mortality data are needed to assess the impact of programs as modelled data cannot separate real changes from changing modelling assumptions. Mortality remains central to burden measures as it comprises most premature deaths and disability in LMICs, and can be measured reliably at low-cost.^15,22^ Disability measurement is less certain, and has far more measurement error than vital status and causes of death.

A practicable solution is to build large, simple nationwide mortality systems that capture a random sample of all deaths, such as the Registrar General of India’s Million Death Study (MDS).^22^ The MDS yielded major findings that substantially changed previous estimates of mortality and the relevance of particular risk factors. For example, it showed that India had only about 0.1 million premature HIV deaths in 2005, about a quarter of the total estimated by WHO models, but that India had far more deaths from malaria than the WHO had estimated. The MDS was able to directly document that a Government of India program has helped reduce child deaths by about 1 million over the past decade, including a 90% reduction in measles mortality.^23^ The Gates Foundation and the Canadian government are investing in nationwide mortality surveillance in African countries. However, further expansion is required. A “25 by 25” goal is desirable. This would aim for 25 major LMICs to have reliable, nationally representative cause of death reporting by 2025.

Dean Jamison’s extraordinary vision of DCP and the GBD has transformed global health. With further practical improvements, the relevance of DCP evidence generation and application over the next 25 years could be as important as in the first 25 years.^12^

## Ethical issues

Not applicable.

## Competing interests

Author declares that he has no competing interests.

## Author’s contribution

PJ is the single author of the paper.
